# The dominant negative ARM domain uncovers multiple functions of PUB13 in *Arabidopsis* immunity, flowering, and senescence

**DOI:** 10.1093/jxb/erv148

**Published:** 2015-04-11

**Authors:** Jinggeng Zhou, Dongping Lu, Guangyuan Xu, Scott A. Finlayson, Ping He, Libo Shan

**Affiliations:** ^1^Institute for Plant Genomics and Biotechnology, Texas A&M University, College Station, TX 77843, USA; ^2^Department of Biochemistry and Biophysics, Texas A&M University, College Station, TX 77843, USA; ^3^Molecular and Environmental Plant Sciences, Texas A&M University, College Station, TX 77843, USA; ^4^Department of Soil and Crop Sciences, Texas A&M University, College Station, TX 77843, USA; ^5^Department of Plant Pathology and Microbiology, Texas A&M University, College Station, TX 77843, USA

**Keywords:** Dominant negative, flowering, phosphorylation, plant–microbe interactions, receptors, senescence, ubiquitination.

## Abstract

Regulating the intensity and duration of immune responses is crucial to combat infections without deleterious side effects. *Arabidopsis* FLS2, the receptor for bacterial flagellin, activates immune signalling by association with its partner BAK1. Upon flagellin (flg22) perception, the plant U-box E3 ubiquitin ligases PUB12 and PUB13 complex with FLS2 in a BAK1-dependent manner, and ubiquitinate FLS2 for protein degradation, thereby down-regulating flagellin signalling. Domain deletion analysis indicates that the ARM domain of PUB13 interacts with the FLS2–BAK1 complex and is phosphorylated by BAK1. Overexpression of the PUB13 ARM domain alone inhibits flg22-induced FLS2–PUB13 association and PUB12/13-mediated FLS2 ubiquitination and degradation in *Arabidopsis*, suggesting that ectopic expression of the ARM domain *in planta* generates a dominant negative effect via blocking the ubiquitination activity. Similar to the *pub12pub13* double mutant, transgenic plants expressing the PUB13 ARM domain display enhanced immune responses compared with wild-type plants. Moreover, PUB13ARM transgenic plants and the *pub12pub13* mutant are more sensitive to stress-induced leaf senescence accompanied by elevated expression of stress-induced senescence marker genes. The resemblance between PUB13ARM transgenic plants and the *pub12pub13* mutant provides genetic evidence that ectopic expression of the PUB ARM domain serves as an alternative approach to dissect the overlapping functions of closely related *PUB* genes.

## Highlight

Ectopic expression of the PUB13 ARM domain *in planta* mimics the *pub13* mutant with enhanced immune responses, stress-induced leaf senescence, and early flowering.

## Introduction

Plants and animals rely on the innate immune system to fight against pathogen invasions. The first line of plant immune responses is initiated by detection of the conserved pathogen- or microbe-associated molecular patterns (PAMPs or MAMPs) through pattern recognition receptors (PRRs) (Macho and Zipfel, 2014). *Arabidopsis* flagellin-sensing 2 (FLS2), a plasma membrane-localized receptor-like kinase (RLK), is a PRR of bacterial flagellin (Gomez-Gomez and Boller, 2000). A 22 amino acid peptide corresponding to the N-terminus of flagellin, flg22, is perceived by FLS2 and induces FLS2 association with another plasma membrane-localized RLK, BAK1, which was originally identified as the plant growth hormone brassinosteroid (BR) receptor BRI1-associated kinase (Li *et al.*, 2002; Nam and Li, 2002; Chinchilla *et al.*, 2007; Heese *et al.*, 2007). BAK1 is required for multiple MAMP-mediated responses and heterodimerizes with other PRRs, including bacterial elongation factor-Tu (EF-Tu) receptor EFR (Roux *et al.*, 2011), and *Arabidopsis* Pep1 receptor AtPEPR1 (Postel *et al.*, 2010). Upon perception of flg22, *Botrytis*-induced kinase 1 (BIK1), a receptor-like cytoplasmic kinase in the FLS2–BAK1 complex, is rapidly phosphorylated by BAK1, and subsequently released from the FLS2–BAK1 complex (Lu *et al.*, 2010; Zhang *et al.*, 2010; Lin *et al.*, 2014). Perception of different MAMPs often elicits a series of overlapping immune responses, including membrane depolarization, reactive oxygen species (ROS) burst, cytoplasmic calcium transients, activation of Ca^2+^-dependent protein kinases (CDPKs) and mitogen-activated protein kinase (MAPK) cascades, ethylene production, transcriptional reprograming, callose deposition, and stomatal closure (Zhang and Zhou, 2010; Wu *et al.*, 2014).

PRR activation and attenuation are also regulated by protein ubiquitination, endocytosis, and degradation (Geldner and Robatzek, 2008; Li *et al.*, 2014). FLS2 has been shown to translocate into intracellular vesicles within 30min of flg22 treatment (Robatzek *et al.*, 2006; Beck *et al.*, 2012). The non-activated FLS2 constitutively recycles between the plasma membrane and early endosome, probably regulating the abundance of receptor at the plasma membrane and maintaining a constant pool of signalling receptor (Beck *et al.*, 2012). Upon flg22 perception, FLS2 enters a distinct endocytic trafficking pathway followed by sorting into the vacuole for degradation (Beck *et al.*, 2012; Choi *et al.*, 2013). As an important post-translational modification process, protein ubiquitination often marks target proteins for degradation through the vacuole or proteasome, or regulates endosomal sorting of plasma membrane-localized proteins (Vierstra, 2009). The ubiquitination pathway directs covalent conjugation of conserved ubiquitin molecules to specific protein substrates through a stepwise reaction mediated by three distinct classes of enzymes, ubiquitin-activating enzyme (E1), ubiquitin-conjugating enzyme (E2), and ubiquitin-protein ligase (E3) (Dreher and Callis, 2007; Vierstra, 2009). The substrate specificity is dictated by ubiquitin E3 ligases, which are broadly classified into three groups: HECT (homologous to E6-AP C-terminus), RING finger, and U-box (Yee and Goring, 2009). It was previously found that two closely related plant U-box (PUB) E3 ubiquitin ligases PUB12 and PUB13 interact with BAK1, and are recruited to FLS2 upon flg22 perception. BAK1 directly phosphorylates PUB12 and PUB13, and is indispensable for FLS2 and PUB12/13 association. PUB12 and PUB13 polyubiquitinate FLS2 and lead to flg22-induced FLS2 degradation, thereby negatively regulating flg22 signalling (Lu *et al.*, 2011). Interestingly, the *pub13* mutant displays certain autoimmune responses, such as spontaneous cell death and accumulation of hydrogen peroxide and the plant defence hormone salicylic acid (SA), when grown under long-day (LD, 16h of light/8h of dark) and high light (250 μE m^–2^ s^–1^) conditions with high humidity (95% relative humidity) treatment (Li *et al.*, 2012). PUB13 also negatively regulates flowering time, suggesting a broad role for PUB13 in controlling plant immunity and growth (Li *et al.*, 2012).

Several PUBs have been implicated in plant defence and cell death control (Craig *et al.*, 2009; Trujillo and Shirasu, 2010). Two Avr9/Cf9 rapidly elicited (ACRE) genes, *ACRE74 (CMPG1*) and *ACRE276 (PUB17*), are required for programmed cell death (PCD) and plant defence in tobacco, tomato, and *Arabidopsis* (Gonzalez-Lamothe *et al.*, 2006; Yang *et al.*, 2006). In contrast, rice Spotted leaf 11 (SPL11) acts as a negative regulator of plant PCD and pathogen resistance (Zeng *et al.*, 2004), and three closely related *Arabidopsis* U-box E3 ubiquitin ligases, PUB22, PUB23, and PUB24, also negatively regulate PAMP-triggered immunity (PTI) signalling (Trujillo *et al.*, 2008). PUB22 interacts with and ubiquitinates Exo70B2, a subunit of the exocyst complex that mediates vesicle tethering during exocytosis (Stegmann *et al.*, 2012). Exo70B2 is required for full activation of multiple PAMP-triggered responses and resistance against different pathogen infections. Perception of flg22 stabilizes PUB22 and promotes PUB22-mediated ubiquitination and degradation of Exo70B2 via the 26S proteasome, thereby attenuating flg22-mediated signalling (Stegmann *et al.*, 2012).

The largest class of PUBs in *Arabidopsis* is the ARMADILLO (ARM) repeat domain-containing PUB proteins (Azevedo *et al.*, 2001; Yee and Goring, 2009). The ARM repeat domain is composed of multiple 42 amino acid ARM repeats with significant sequence divergence but a highly conserved right-handed superhelix of α-helices that is predicted to form a specific protein interaction domain (Samuel *et al.*, 2006). In this study, a series of domain deletion and truncation studies of PUB12 and PUB13 were performed. It is shown that the ARM domain of PUB13 interacts with BAK1 and is phosphorylated by BAK1. Ectopic expression of the PUB13 ARM domain inhibits flg22-induced FLS2–PUB13 association and PUB12/13-mediated FLS2 ubiquitination and degradation in *Arabidopsis*. Similar to the *pub12pub13* double mutant, transgenic plants expressing the PUB13 ARM domain display enhanced immune responses compared with wild-type (WT) plants. Apparently, ectopic expression of the protein–protein interacting ARM domain creates a dominant negative effect and interferes with the endogenous PUB functions, implying a potential alternative to dissect the overlapping functions of multiple closely related *PUB* genes. Interestingly, similarly to the *pub12pub13* double mutant, PUB13ARM transgenic plants showed enhanced dark-induced senescence accompanied by elevated expression of the stress-induced senescence marker genes, senescence-associated gene 13 (*SAG13*) and *SAG14*.

## Materials and methods

### Plant materials and growth conditions

The *pub12* and *pub13* single mutant and *pub12/13* double mutant plants were described previously (Lu *et al.*, 2011). *Arabidopsis* plants were grown in soil (Metro Mix 360) in a growth chamber with 23 °C, 65% relative humidity, 75 μE m^–2^ s^–1^ light, and a photoperiod of 12h light/12h dark for 4 weeks before protoplast isolation and disease assays. For flowering time, plants were grown under 23 °C, 65% relative humidity, 75 μE m^–2^ s^–1^ light, and a photoperiod of 16h light/8h dark. To grow *Arabidopsis* seedlings on medium, the seeds were surface-sterilized with 50% bleach for 15min, washed with sterilized ddH_2_O, and then placed on plates with half-strength Murashige and Skoog medium (1/2 MS) containing 0.5% sucrose, 0.8% agar, and 2.5 mM MES at pH 5.7. The plates were first stored at 4 °C for 3 d in the dark for seed stratification, and then moved to the growth chamber set to the same parameters as the soil-grown plants.

### Plasmid construction and generation of transgenic plants

The constructs of *BAK1*, *FLS2*, and *PUB13* in the plant expression vector (pHBT) or protein expression vector (pMAL or pGST) were reported previously (Lu *et al.*, 2010, 2011). The different *PUB13* domain truncations were cloned into the plant expression vector, protein expression vector, or modified bimolecular fluorescence complementation (BiFC) vector with PCR amplification using the primers listed in Supplementary Table S1 available at *JXB* online. The *35S::FLS2-LUC* construct was made by subcloning the full-length *FLS2* gene into the plant expression vector pHBT fused with a *Luciferase* gene. The *PUB13ARM* was subcloned into a modified plant transformation binary vector *pCB302* derivative under the control of the *35S* promoter with an HA epitope tag. The transgenic plants were generated by *Agrobacterium tumefaciens*-mediated transformation in *Arabidopsis* accession Col-0 plants. The transgenic plants were screened with the herbicide BASTA (resistance conferred by the binary vector) and confirmed by western blotting with an α-HA antibody. The homozygous lines were selected based on the survival ratio of T_2_ and T_3_ generation plants after BASTA spray.

### *In vitro* pull-down assay

Maltose-binding protein (MBP), MBP–BAK1CD, MBP–BAK1CDKm, MBP–FLS2CD, glutathione *S*-transferase (GST), GST–PUB13, and GST–PUB13ARM proteins were individually expressed in *Escherichia coli* BL21 strain and purified with a standard amylose or glutathione agarose-based procedure. About 10 μg of MBP, MBP–BAK1CD, or MBP–FLS2CD proteins were incubated with 5 μl of pre-washed amylose beads in 1ml of pull-down buffer (20mM TRIS-HCl, pH 7.5, 1mM β-mercaptoethanol, 3mM EDTA, 150mM NaCl, and 1% NP-40) for 30min at 4 °C with gentle shaking. The beads were harvested by centrifugation at 2000rpm for 1min and then inoculated with 10 μg of GST or GST-tagged proteins in 1ml of pull-down buffer for 1h at 4 °C with gentle shaking. The beads were harvested and washed three times with 1ml of pull-down buffer and once with 1ml of 50mM TRIS-HCl, pH 7.5. Bound proteins were released from beads by boiling in 20 μl of 2× SDS–PAGE sample loading buffer and analyaed by western blot with an α-GST antibody.

### BiFC assay

Protoplast isolation and transfection were performed as described (He *et al.*, 2007). *Arabidopsis* protoplasts were co-transfected with various BiFC constructs as indicated in the figures. Fluorescence signals in protoplasts were visualized under a confocal microscope (Olympus FV1000) 18h after transfection. The following are the filter sets used for excitation (Ex) and emission (Em): yellow fluorescent protein (YFP), 515nm (Ex)/520–550nm (Em); chlorophyll, 488nm (Ex)/560–650nm (Em); bright field, 633nm. Images were captured in a multichannel mode, analysed, and processed with an OLYMPUS FLUOVIEW Version 3.0 Viewer.

### Co-immunoprecipitation and *in vivo* ubiquitination assays

The total proteins from 2×10^5^ transfected protoplasts were isolated with 0.5ml of extraction buffer (10mM HEPES, pH 7.5, 100mM NaCl, 1mM EDTA, 10% glycerol, 0.5% Triton X-100, and 1× protease inhibitor cocktail from Roche). The samples were vortexed vigorously for 30 s, and then centrifuged at 13 000rpm for 10min at 4 °C. The supernatant was inoculated with α-FLAG agarose beads for 2h at 4 °C with gentle shaking. The beads were collected and washed three times with washing buffer (10mM HEPES, pH 7.5, 100mM NaCl, 1mM EDTA, 10% glycerol, 0.1% Triton X-100, and 1× protease inhibitor cocktail) and once more with 50mM TRIS-HCl, pH 7.5. Bound proteins were released from the beads by boiling in SDS–PAGE sample loading buffer and analysed by western blot with an α-HA antibody.

### *In vitro* phosphorylation, immunocomplex kinase and *in vivo* MAPK assays

For *in vitro* kinase assay, reactions were performed in 30 μl of kinase buffer [20mM TRIS-HCl, pH 7.5, 10mM MgCl_2_, 5mM EGTA, 100mM NaCl, and 1mM dithiothreitol (DTT)] containing 10 μg of fusion proteins with 0.1mM cold ATP and 5 μCi of [γ-^32^P]ATP at room temperature for 3h with gentle shaking. The reactions were stopped by adding 4× SDS loading buffer. The phosphorylation of fusion proteins was analysed by autoradiography after separation by 12% SDS–PAGE.

For immunocomplex kinase assay, ten 2-week-old seedlings were ground with 1ml of IP buffer (50mM TRIS-HCl, pH 7.5, 150mM NaCl, 5mM EDTA, 1mM DTT, 2mM NaF, 2mM Na_3_VO_3_, 1% Triton, and protease inhibitor cocktail). The samples were vortexed vigorously for 30 s, and then centrifuged at 13 000rpm for 10min at 4 °C. The supernatant was incubated with α-MPK antibody for 2h and then with protein-G–agarose beads for another 2h at 4 °C with gentle shaking. The beads were harvested and washed once with IP buffer and once with kinase buffer. The kinase reactions were performed in 20 μl of kinase buffer with 2 μg of MBP as substrate, 0.1mM cold ATP, and 5 μCi of [γ-^32^P]ATP at room temperature for 1h with gentle shaking. The phosphorylation of MBP proteins was analysed by autoradiography after separation by 15% SDS–PAGE.

For detecting MAPK activity *in vivo*, 2-week-old seedlings grown on 1/2 MS medium were transferred to water overnight and then treated with 100nM flg22 or H_2_O for the times indicated and frozen in liquid nitrogen. The seedlings were homogenized in IP buffer and an equal amount of total protein was electrophoresed by 10% SDS–PAGE. An α-pERK antibody (1:2000) (Cell Signaling) was used to detect the phosphorylation status of MPK3 and MPK6 with an immunoblot.

### *In vitro* ubiquitination assay

The *in vitro* ubiquitination assay was performed as described with some modifications (Lu *et al.*, 2011). The reactions contain 1 μg of purified MBP–FLS2CD, 1 μg of purified His_6_-E1 (AtUBA1), 1 μg of purified His_6_-E2 (AtUBC8), 1 μg of His_6_-ubiquitin (Boston Biochem), 1 μg of purified GST–PUB, and 1–16 μg of purified GST or GST–ARM with 6 μl of 5× ubiquitination buffer (0.1M TRIS-HCl, pH 7.5, 25mM MgCl_2_, 2.5mM DTT, 10mM ATP) to a final volume of 30 μl. The reactions were incubated at 30 °C for 2h, and then stopped by adding SDS sample buffer and boiled at 100 °C for 5min. The samples were then separated by 7.5% SDS–PAGE and the ubiquitinated substrates were detected by western blot analysis.

### Measurement of ROS production

Four leaves of each of six 5-week-old *Arabidopsis* plants were excised into leaf discs of 0.25cm^2^, following an overnight incubation in a 96-well plate with 100 μl of H_2_O to eliminate the wounding effect. H_2_O was replaced by 100 μl of reaction solution containing 50 μM luminol and 10 μg ml^–1^ horseradish peroxidase (Sigma) supplemented with 100nM flg22. The luminescence was measured with a luminometer (Perkin Elmer, 2030 Multilabel Reader, Victor X3) immediately after adding the solution, with a 2min interval reading time for a period of 60min. The measurement value of ROS production from 24 leaf discs per treatment was indicated as the mean of RLU (relative light units).

### Quantitative reverse transcription–PCR (qRT-PCR) analysis

Total RNA was isolated from leaves or seedlings after treatment with TRIzol Reagent (Invitrogen). cDNA was synthesized from 1 µg of total RNA with oligo(dT) primer and reverse transcriptase (New England BioLabs). Real-time RT–PCR analysis was carried out using iTaq SYBR green Supermix (Bio-Rad) supplemented with ROX in an ABI GeneAmp PCR System 9700. The expression of immunity-related genes was normalized to the expression of UBQ10. The primer sequences for RT–PCR are listed in Supplementary Table S1 at *JXB* online.

### Callose deposition

Three 2-week-old seedlings were incubated with 500nM flg22 for 12h at room temperature. Seedlings were immediately cleared in alcoholic lactophenol [95% ethanol:lactophenol (phenol:glycerol:lactic acid:H_2_O 1:1:1:1)=2:1] overnight. Samples were subsequently rinsed with 50% ethanol and H_2_O. Cleared leaves were stained with 0.01% aniline blue in 0.15M phosphate buffer (pH 9.5) and the callose deposits were visualized under a UV filter using a fluorescence microscope. Callose deposits were counted using ImageJ 1.43U software (http://rsb.info.nih.gov/ij/). The number of deposits was expressed as the mean of six different leaf areas with standard error.

### Pathogen infection assays

*Pseudomonas syringae* pv. *tomato* (*Pst*) DC3000 and *P. syringae* pv. *maculicola* (*Psm*) ES4326 strains were cultured overnight at 28 °C in KB medium with 50 μg ml^–1^ rifampicin for DC3000 and 50 μg ml^–1^ streptomycin for ES4326. Bacteria were collected, washed, and diluted to the desired density with H_2_O. Four-week-old *Arabidopsis* leaves were infiltrated with bacteria at a concentration of 5×10^5^ cfu ml^–1^ using a needleless syringe. To measure bacterial growth, two leaf discs were ground in 100 μl of H_2_O and serial dilutions were plated on KB medium with appropriate antibiotics. Bacterial colony-forming units (cfu) were counted 2 d after incubation at 28 °C. Each data point is shown as triplicates.

*Botrytis cinerea* BO5 was grown on potato dextrose agar (Difco, USA) and was incubated at room temperature. Conidia were re-suspended in distilled water and the spore concentration was adjusted to 10^5^ spores ml^–1^. Gelatin (0.5%) was added to the conidial suspension before inoculation. The detached *Arabidopsis* leaves were laid on a plastic tray with wet paper at the bottom and 10 μl of spores were dropped onto the centre of each leaf. The tray was covered with a dome to maintain high humidity, and disease development was monitored over a period of 3 d.

### SA measurement

For SA measurements, 4-week-old plants were infested with *Pst* at a concentration of 5×10^5^ cfu ml^–1^, or water as a mock control. Three days later, leaves were harvested and ground in liquid nitrogen. SA was quantified using the method described previously (Lei *et al.*, 2014) with the following modifications. During solvent partitioning, the pH of the aqueous solution was first adjusted to pH 8.0 and then to pH 6.0, and the mass spectrometer was operated in SIM mode only.

### Trypan blue and DAB staining

For trypan blue staining, the leaves of 4-week-old plants were excised and subsequently immersed in boiled lactophenol (lactic acid:glycerol:liquid phenol:distilled water 1:1:1:1) solution with 0.25mg ml^–1^ trypan blue for 1min. The stained leaves were destained with 95% ethanol/lactophenol solution, and washed with 50% ethanol. For DAB staining, the leaves of 4-week-old plants were immersed in 1mg ml^–1^ DAB (3,3’-diaminobenzidine) (pH 3.8) solution with low vacuum pressure for 30min, followed by an overnight incubation at room temperature in the dark. The stained leaves were fixed and cleared in alcoholic lactophenol (95% ethanol:lactic acid:phenol 2:1:1) at 65 °C, and rinsed once with 50% ethanol and twice with H_2_O. The destained leaves were subjected to microscopic observation.

## Results

### PUB13 ARM domain interacts with and is phosphorylated by BAK1

**Fig. 1.**
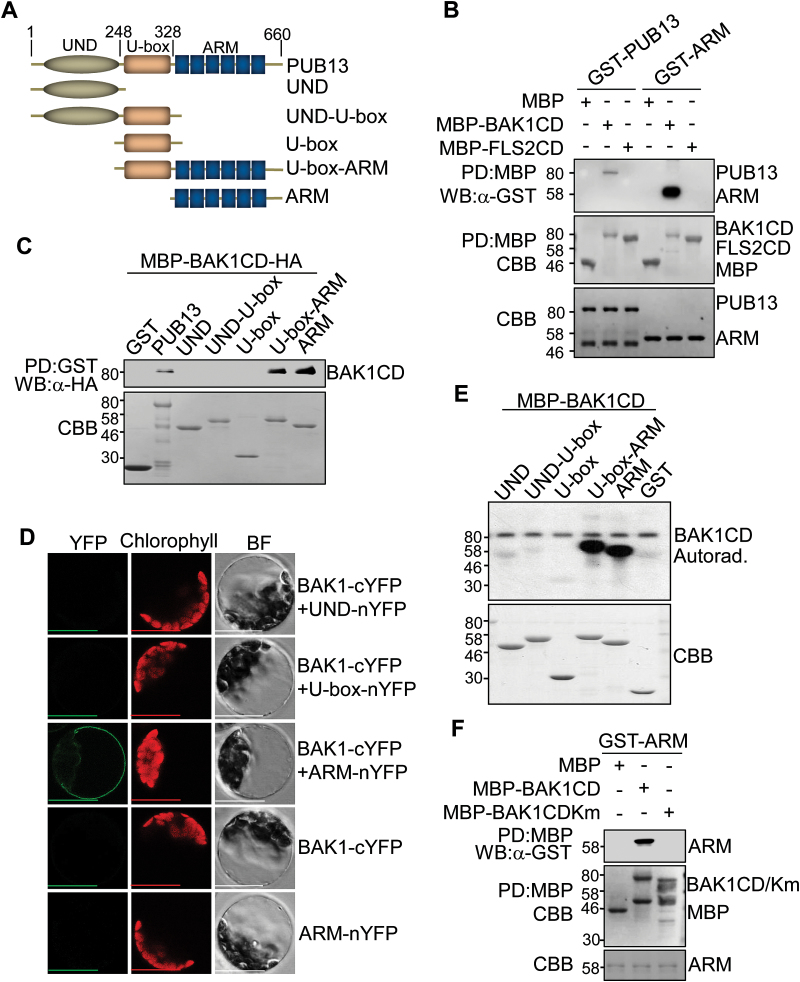
The PUB13 ARM domain interacts with and phosphorylates BAK1. (A) A schematic protein domain structure of PUB13. PUB13 contains a U-box N-terminal domain (UND), a U-box domain, and a C-terminal ARMADILLO (ARM) repeat domain. The amino acid position is labelled on the top. The PUB13 truncations used for the following experiments are listed. (B and C) The ARM domain interacts with the BAK1 cytosolic domain (CD) in *in vitro* pull-down (PD) assays. (B) GST-fused PUB13 or ARM proteins were incubated with MBP, MBP–BAK1CD, or MBP–FLS2CD amylose beads, and the beads were collected and washed for western blot (WB) of immunoprecipitated proteins with an α-GST antibody (top gel). (C) MBP–BAK1CD was incubated with GST, GST–PUB13, or various truncated forms of PUB13 proteins with glutathione beads, and the beads were collected and washed for WB of immunoprecipitated proteins with an α-HA antibody (top gel). The protein inputs are indicated by Coomassie Brilliant Blue (CBB) staining. (D) The ARM domain interacts with BAK1 with BiFC assay in *Arabidopsis* protoplasts. Protoplasts were transfected with various BiFC constructs, and the fluorescence was visualized under a confocal microscope. YFP, yellow fluorescent protein signal; Chlorophyll, autofluorescence signal; BF, bright field. Scale bars=50 μm. (E) The ARM domain is phosphorylated by BAK1. GST–PUB13 or various truncated forms of PUB13 proteins (10 μg) were used as substrates and MBP–BAK1CD (1 μg) as the kinase in an *in vitro* kinase assay. Phosphorylation was detected by autoradiography (top panel), and the protein loading is shown by CBB staining (bottom panel). (F) Interaction of PUB13ARM with BAK1 requires BAK1 kinase activity. GST–ARM proteins were incubated with MBP, MBP–BAK1CD, or MBP–BAK1CDKm amylose beads for *in vitro* PD assay. The PD precipitates were examined with α-GST antibody in WB (top panel). The protein inputs are shown by CBB staining (middle and bottom panels). The above experiments were repeated three times with similar results.

PUB13 contains a U-box N-terminal domain (UND), a U-box domain, and a C-terminal ARM repeat domain (Fig. 1A). PUB13 co-immunoprecipitated with BAK1 and FLS2 in *Arabidopsis* (Lu *et al.*, 2011). To test whether PUB13 directly interacts with BAK1 and/or FLS2, an *in vitro* pull-down assay was performed with the cytoplasmic domain of BAK1 (BAK1CD) or FLS2 (FLS2CD) fused to MBP immobilized on amylose–agarose beads as the bait against GST–PUB13 fusion proteins. As shown in Fig. 1B, GST–PUB13 was able to be pulled down by MBP–BAK1CD, but not by MBP–FLS2CD or MBP itself, suggesting a physical interaction between PUB13 and BAK1 but not between PUB13 and FLS2. This is consistent with a previous report showing that PUB13 constitutively associates with BAK1, whereas flg22 induces BAK1-dependent PUB13 and FLS2 association (Lu *et al.*, 2011). It has been suggested that the ARM domain mediates protein–protein interaction whereas the U-box domain confers E3 ubiquitin ligase activity (Samuel *et al.*, 2006). It was further examined whether the ARM domain is sufficient to mediate the PUB13 interaction with BAK1. As shown in Fig. 1B, the ARM domain of PUB13 (GST–ARM) was pulled down by MBP–BAK1CD, indicating that the ARM domain mediates a direct interaction with BAK1. Notably, the ARM domain interaction with BAK1 appears to be much stronger than that of full-length PUB13 (Fig. 1B). Pull-down assays were further performed using different truncations of PUB13 with MBP–BAK1CD. The BAK1 interaction with the U-box-ARM domain is similar to that with the ARM domain, and stronger than that with PUB13, but no interaction was detected with the UND (Fig. 1C). These results suggest that the UND may negatively regulate PUB13–BAK1 interaction. To test the *in vivo* interaction of PUB13ARM and BAK1, BiFC assay was performed. A strong fluorescence signal was detected in the cell periphery with co-transfection of BAK1 fused to the C-terminal half of YFP (BAK1–cYFP) and PUB13ARM fused to the N-terminal half of YFP (ARM–nYFP) in protoplasts, suggesting that PUB13ARM interacts with BAK1 *in vivo* (Fig. 1D). Neither of the individual constructs nor co-transfection of UND–nYFP or U-box–nYFP with BAK1–cYFP emitted YFP signals in protoplasts. PUB13 is a phosphorylation substrate of BAK1 (Lu *et al.*, 2011). The interaction between the ARM domain of PUB12 or PUB13 and BAK1 was confirmed in a co-immunoprecipitation (Co-IP) assay (Supplementary Fig. S1 at *JXB* online). It was determined which domain(s) of PUB13 mediates the phosphorylation by BAK1 by using an *in vitro* kinase assay employing MBP–BAK1CD as a kinase and different truncated versions of PUB13 as substrates in the presence of [γ-^32^P]ATP. Consistent with a previous report (Lu *et al.*, 2011), BAK1CD possesses autophosphorylation activity and could phosphorylate the full-length PUB13 (Supplementary Fig. S2). Apparently, BAK1CD strongly phosphorylated the ARM domain or U-box-ARM domain yet exhibited little phosphorylation activity towards the UND, UND–U-box, or U-box domain (Fig. 1E). The data indicate that BAK1 phosphorylation mainly occurs on the ARM domain of PUB13. Notably, the BAK1CD kinase-inactive mutant (BAK1CDKm), in which Lys317 was mutated to methionine (K317M), was no longer able to interact with PUB13ARM in an *in vitro* pull-down assay (Fig. 1F), indicating that BAK1 kinase activity is essential in mediating its interaction with PUB13.

### Ectopic expression of PUB13ARM blocks PUB13-mediated FLS2 ubiquitination and degradation

**Fig. 2.**
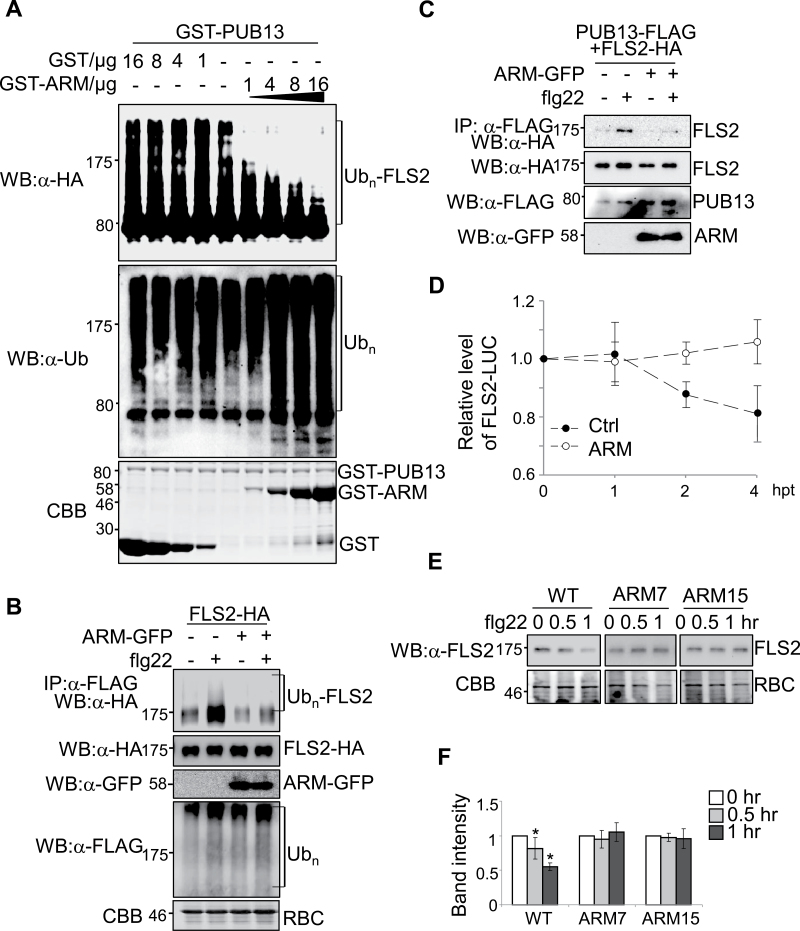
Ectopic expression of PUB13ARM blocks flg22-induced FLS2 ubiquitination and degradation. (A) PUB13ARM blocks PUB13-mediated FLS2 ubiquitination. MBP–FLS2CD-HA was incubated with GST–PUB13 in an *in vitro* ubiquitination reaction in the presence of different amount of proteins of GST (Control) or GST–ARM. The ubiquitination of FLS2CD was detected by α-HA antibody (top panel). The overall ubiquitination was detected by α-ubiquitin (Ub) antibody (middle panel). GST protein loading is shown by Coomassie Brilliant Blue (CBB) staining (bottom panel). (B) Ectopic expression of PUB13ARM blocks flg22-induced FLS2 ubiquitination. *Arabidopsis* protoplasts were transfected with FLAG-Ub, FLS2-HA, and PUB13ARM–GFP or a control vector, and incubated for 10h before 1 μM flg22 treatment for 30min. The ubiquitinated FLS2 was detected with an α-HA antibody western blot (WB) after immunoprecipitation with α-FLAG antibody. The total ubiquitinated proteins were detected by an α-FLAG WB, and the protein levels of FLS2 and ARM were detected by α-HA or α-GFP WB, respectively. Equal protein loading is shown by CBB staining for RuBisCo (RBC). (C) Ectopic expression of PUB13ARM suppresses flg22-induced FLS2–PUB13 association. *Arabidopsis* protoplasts were transfected with FLS2-HA, PUB13-FLAG, and PUB13ARM–GFP or a control vector, and incubated for 10h before 1 μM flg22 treatment for 10min. The association of FLS2–PUB13 was detected by an α-HA WB after α-FLAG immunoprecipitation. The protein levels of FLS2, PUB13, and ARM were detected by α-HA, α-FLAG, or α-GFP WB, respectively. (D) Ectopic expression of PUB13ARM inhibits flg22-induced FLS2–LUC degradation in protoplasts. The 35S::FLS2-LUC was co-transfected with ARM or a control vector in *Arabidopsis* protoplasts. The transfected protoplasts were incubated for 12h before 10 μM cycloheximide treatment for 1h, and then treated with 1 μM flg22 for the indicated times (hpt, hours post flg22 treatment). The UBQ–GUS was included as an internal transfection control. The LUC activity was normalized to GUS activity to reflect the relative protein level of FLS2. (E and F) Stable expression of PUB13ARM in transgenic plants blocks flg22-induced FLS2 degradation. ARM7 and ARM15 are two independent PUB13ARM transgenic lines (ARM7-2 and ARM15-1). Two-week-old seedlings were treated with 1 μM flg22 for 0.5h or 1h. The endogenous FLS2 proteins were detected by α-FLS2 WB (E) and were quantified by the band intensity (F). Equal protein loading is shown by CBB staining for RBC. The data are shown as the mean ±standard error (*n*=3), and the asterisk (*) indicates a significant difference before and after flg22 treatment (*P*<0.05). The above experiments were repeated three times with similar results.

It was further tested whether ectopic expression of the ARM domain *in planta* will generate a dominant negative effect on the function of endogenous PUB13 and its closest homologues, such as PUB12. It was first determined whether excess ARM domain protein could interfere with PUB13 ubiquitination activity towards FLS2CD in an *in vitro* ubiquitination assay. The addition of PUB13ARM proteins into the reactions resulted in a gradually shortened ladder formation of FLS2CD in a dosage-dependent manner (Fig. 2A). Notably, the overall ubiquitination reaction was not affected by PUB13ARM protein as detected with an α-ubiquitin (α-Ub) antibody (Fig. 2A). The effect of ARM protein on FLS2 ubiquitination was further tested with an *in vivo* ubiquitination assay. HA epitope-tagged full-length FLS2 and FLAG epitope-tagged Ub were co-expressed in the presence or absence of GFP-tagged PUB13ARM in *Arabidopsis* protoplasts. The ubiquitinated FLS2 protein was detected with an α-HA western blot upon immunoprecipitation with α-FLAG antibody. As reported, flg22 treatment induced FLS2 ubiquitination as shown by the enhanced ladder-like smear formation (Fig. 2B). Ectopic expression of PUB13ARM–green fluorescent protein (GFP) largely diminished flg22-induced ladder formation, suggesting compromised flg22-induced FLS2 ubiquitination (Fig. 2B). Thus, the PUB13 ARM domain is able to interfere with the ubiquitination activity of full-length PUB13 towards FLS2 in both *in vitro* and *in vivo* ubiquitination assays.

Next, it was tested whether PUB13ARM interferes with flg22-induced FLS2–PUB13 association by Co-IP assay (Fig. 2C). FLAG-tagged PUB13 was co-expressed with HA-tagged FLS2, and FLS2 was detected in the precipitates with an α-HA western blot upon immunoprecipitation with α-FLAG antibody. Apparently, ectopic expression of PUB13ARM–GFP reduced flg22-induced PUB13–FLS2 association (Fig. 2C). The prolonged flg22 treatment induces FLS2 degradation (Lu *et al.*, 2011). To quantify the FLS2 degradation rate, the firefly luciferase gene (*LUC*) was fused to the C-terminus of the full-length *FLS2* gene under the control of the constitutive *Cauliflower mosaic virus* (CaMV) *35S* promoter. The resulting construct *35S::FLS2-LUC* was transfected into *Arabidopsis* protoplasts and the luciferase activity was monitored upon flg22 treatment (Fig. 2D). A reduction of luciferase activity was observed 2h and 4h after flg22 treatment in the vector control transfection, indicating the occurrence of flg22-induced FLS2–LUC degradation (Fig. 2D). However, ectopic expression of PUB13ARM ameliorated flg22-induced degradation of FLS2–LUC luciferase activity, suggesting that PUB13ARM blocks flg22-induced FLS2 degradation (Fig. 2D). Transgenic plants carrying HA-tagged PUB13ARM under the control of the CaMV *35S* promoter were further generated in the WT *Arabidopsis* Col-0 ecotype. Two independent transgenic lines, 7-2 and 15-1, with moderate protein expression levels of PUB13ARM, were selected for phenotypic and molecular analyses (Supplementary Fig. S3A at *JXB* online). The PCR analysis indicated that the transgenes did not disrupt the endogenous *PUB13* gene (Supplementary Fig. S3B). To examine flg22-induced endogenous FLS2 protein degradation, the FLS2 protein level in these transgenic plants was monitored with α-FLS2 antibody (Fig. 2E, F). In WT plants, the protein level of FLS2 was reduced by ~20% and 50% at 30min and 60min after flg22 treatment, respectively. However, no significant change in the FLS2 protein level was detected in the two lines of PUB13ARM transgenic plants upon flg22 treatment (Fig. 2E, F), which is consistent with the result of FLS2–LUC reporter assays in protoplasts (Fig. 2D). Notably, it appears that the degradation rate of FLS2–LUC in the protoplast-based assay was slower than that of endogenous FLS2 in the seedling assay, probably due to the different protein expression level of FLS2. Taken together, ectopic expression of PUB13ARM blocked flg22-induced FLS2 ubiquitination and degradation in *Arabidopsis*.

### The PUB13ARM transgenic plants display elevated flg22-mediated immune responses

**Fig. 3.**
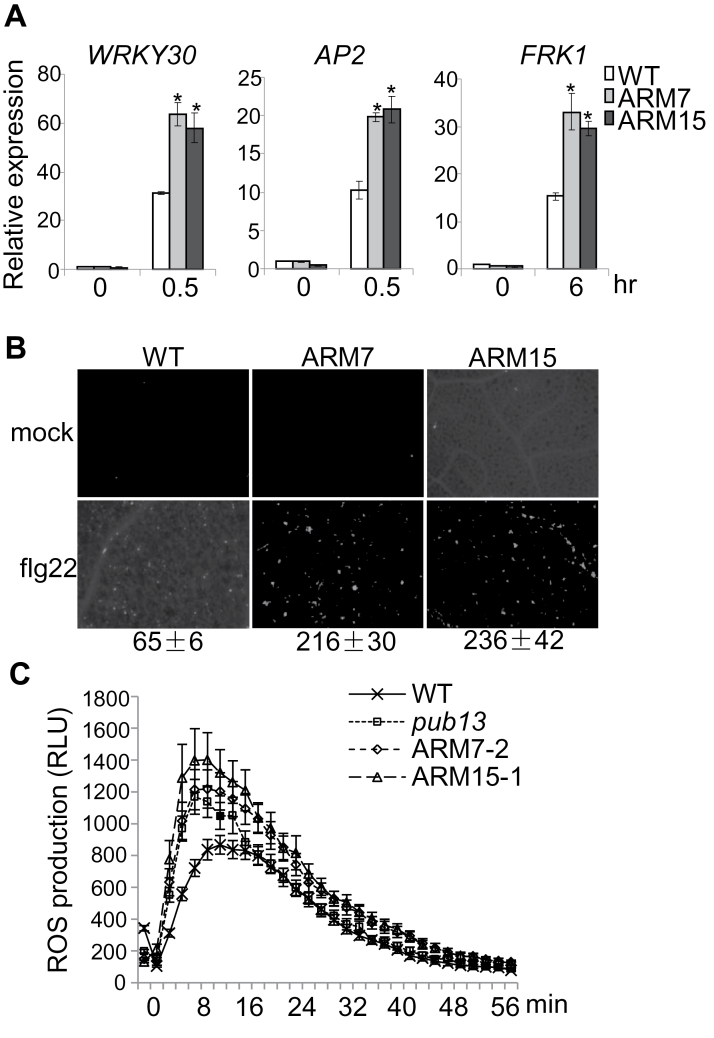
Elevated flg22-mediated immune responses in PUB13ARM transgenic plants. (A) Expression of flg22-induced genes in PUB13ARM transgenic plants. Real-time RT–PCR analysis of 2-week-old seedlings after 0.5h or 6h treatment with 100nM flg22. The expression of *WRKY30*, *AP2*, and *FRK1* was normalized to the expression of *UBQ10*. The data are shown as the mean ±SE (*n*=3), and asterisks (*) indicate a significant difference between WT and PUB13ARM transgenic plants (*P*<0.05). (B) Callose deposition in PUB13ARM transgenic plants. Two-week-old seedlings were collected for aniline blue staining 12h after treatment with 500nM flg22. Callose deposits were counted using ImageJ 1.43U software (http://rsb.info.nih.gov/ij/). The data are shown as the mean ±SE (*n*=6). (C) ROS production in PUB13ARM transgenic plants. Leaf discs from 5-week-old plants were treated with 100nM flg22, and ROS production was detected at the indicated time points. The data are shown as the mean ±SE from 24 leaf discs. The above experiments were repeated three times with similar results.

The *pub12pub13* (*pub12/13*) double mutant showed elevated flg22-mediated defence gene activation, callose deposition, and ROS production (Lu *et al.*, 2011). These immune responses were examined in PUB13ARM transgenic plants. qRT–PCR analysis showed that flg22-mediated induction of the defence genes *WRKY30* (*At5g24110*), *AP2* (*At3g23230*), and *FRK1* (*At2g19190*) was increased ~2-fold in PUB13ARM transgenic plants compared with that in WT plants (Fig. 3A). Similar to the *pub12/13* mutant plants, PUB13ARM transgenic plants accumulated >3-fold more callose deposits than WT plants 12h after flg22 treatment (Fig. 3B). In addition, the peak of flg22-induced ROS production in PUB13ARM transgenic plants was ~50% higher than that in WT plants (Fig. 3C). Taken together, the data indicate that flg22-induced immune responses were elevated in PUB13ARM transgenic plants compared with WT plants.

### PUB13ARM transgenic plants display elevated flg22-induced MAPK activation

**Fig. 4.**
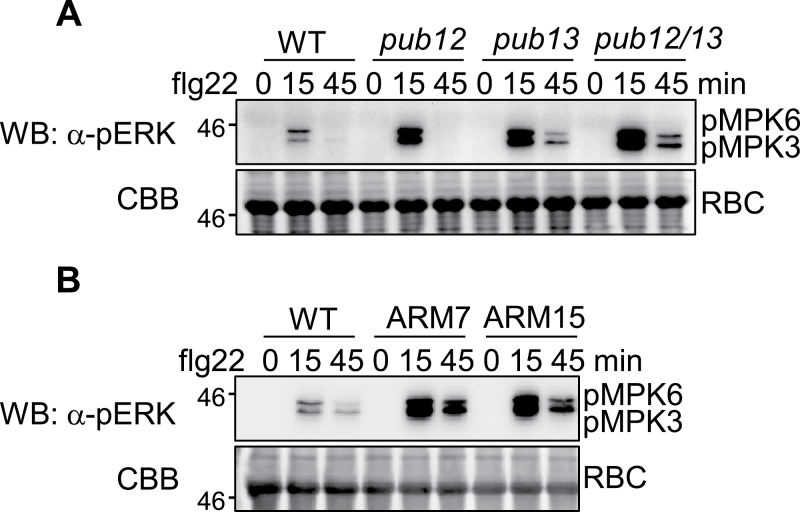
PUB12/13 negatively regulate MAPK activation. (A and B) Enhanced MAPK activity in *pub12/13* mutant and PUB13ARM transgenic plants as detected by α-pERK antibody. Two-week-old seedlings were treated with 100nM flg22 for 15min or 45min. Phosphorylated MPK3 (pMPK3) and MPK6 (pMPK6) were detected by α-pERK western blot (WB). Protein loading is shown by Coomassie Briliant Blue (CBB) for RBC. The above experiments were repeated three times with similar results.

Activation of MAPKs is one of the earliest signalling events following MAMP recognition (Meng and Zhang, 2013). It remains unknown whether PUB13 and its closest homologue PUB12 also regulate flg22-induced MAPK activation. Thus flg22-mediated activation of MAPKs was first examined with α-pERK antibody that cross-reacts with phosphorylated *Arabidopsis* MPK3 and MPK6. MPK3 and MPK6 were activated 15min after flg22 treatment in WT plants, whereas the activation was enhanced in *pub12*, *pub13*, and *pub12/13* mutant seedlings compared with WT plants (Fig. 4A). An immunocomplex kinase assay was further carried out to determine the activation of endogenous MPK3 and MPK4. The MPK3 and MPK4 proteins were immunoprecipitated with α-MPK3 or α-MPK4 antibody from WT or *pub12/13* mutant seedlings treated or not with flg22, and subjected to an *in vitro* kinase assay using myelin basic protein as a substrate in the presence of [γ-^32^P]ATP. Consistent with the detection by α-pERK antibody, flg22 treatment elicited a stronger activation of MPK3 and MPK4 in *pub12/13* mutants compared with WT plants (Supplementary Fig. S4A, B at *JXB* online). Importantly, two independent lines of PUB13ARM transgenic plants also exhibited enhanced flg22-mediated MPK3 and MPK6 activation compared with WT plants (Fig. 4B). Notably, the elevated MAPK activation in PUB13ARM transgenic plants resembled that detected in the *pub12/13* double mutant and was more pronounced than that in *pub12* or *pub13* single mutants, suggesting that ectopic expression of PUB13ARM may interfere with both *PUB12* and *PUB13* functions.

### PUB13ARM transgenic plants show enhanced disease resistance

**Fig. 5.**
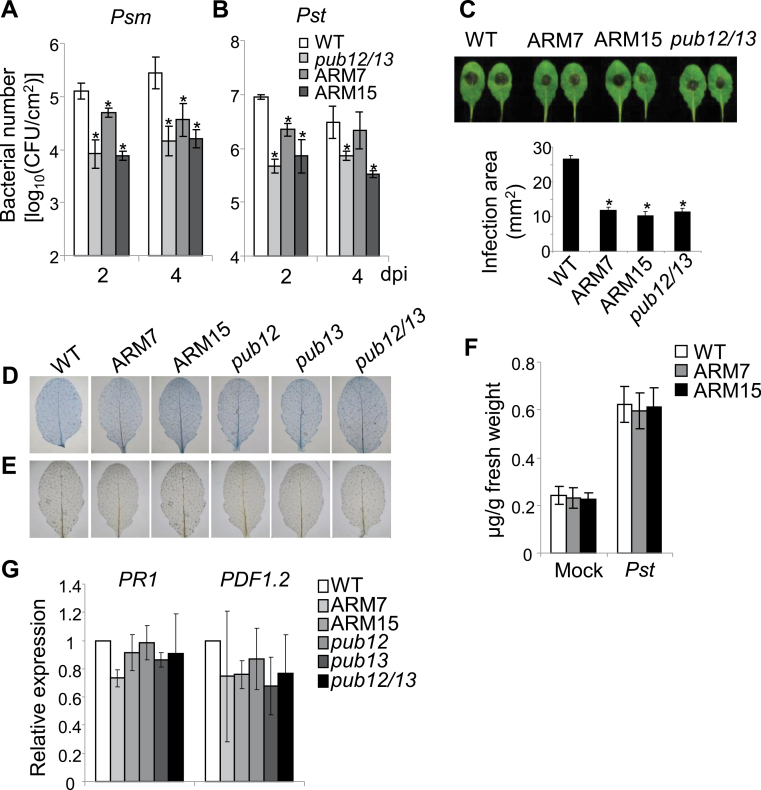
Enhanced disease resistance in PUB13ARM transgenic plants. (A and B) Growth of *Pseudomonas syringae* in PUB13ARM transgenic plants. Four-week-old *Arabidopsis* leaves were hand-inoculated with *P. syringae* pv. *maculicola* ES4326 or *P. syringae* pv. *tomato* DC3000 strain at 5×10^5^ cfu ml^–1^. Bacterial counting was performed 2 d and 4 d post-inoculation (dpi). An asterisk (*) indicates a significant difference (*P*<0.05) between WT and PUB13ARM transgenic plants. (C) Lesion development of *Botrytis cinerea* in PUB13ARM transgenic plants. Five-week-old plants were infected with *B. cinerea* BO5 at the concentration of 10^5^ spores ml^–1^. Disease symptoms were recorded and the area of infection was measured 3 dpi. An asterisk (*) indicates a significant difference with *P*<0.05 between WT and PUB13ARM transgenic plants. (D) Cell death and (E) H_2_O_2_ accumulation in the leaves of 4-week-old plants were examined by trypan blue and DAB staining respectively. (F) SA levels in WT and PUB13ARM transgenic plants. Four-week-old *Arabidopsis* leaves were hand-inoculated with *Pst* DC3000 strain at 5×10^5^ cfu ml^–1^. SA was analysed 3 dpi. The data are shown as the mean ±SE (*n*=4). (G) The expression of *PR1* and *PDF1.2* in 4-week-old plants was normalized to the expression of *UBQ10*. The data are shown as the mean ±SE (*n*=3). The plants were grown under 23 °C, 65% relative humidity, 75 μE m^–2^ s^–1^ light, and a photoperiod of 12h light/12h dark. The above experiments were repeated three times with similar results.

The *pub12/13* mutant is more resistant to infections by *Psm* ES4326 and *Pst* DC3000 compared with WT plants (Lu *et al.*, 2011). It was tested whether PUB13ARM transgenic plants were also more resistant to bacterial infections. At 2 d or 4 d after inoculation, the *in planta* bacterial population of *Psm* ES4326 in two independent lines of PUB13ARM transgenic plants or the *pub12/13* mutant was ~5- to 10-fold lower than that in WT plants (Fig. 5A). A similar result was observed with *Pst* DC3000 infection (Fig. 5B). Thus, similar to the *pub12/13* double mutant, PUB13ARM transgenic plants are more resistant to *Psm* and *Pst* infections than WT plants. In addition, the infection by the necrotrophic pathogen *Botrytis cinerea* BO5 was also examined in *pub12/13* mutant and PUB13ARM transgenic plants. Three days after infection, leaves detached from WT plants formed a large lesion at the infection sites. In contrast, the lesion area on the leaves of *pub12/13* mutant and PUB13ARM transgenic plants was ~50% smaller than that on WT leaves (Fig. 5C), suggesting that both *pub12/13* mutant and PUB13ARM transgenic plants were more resistant to necrotrophic *Botrytis* infection than WT plants in the growth conditions used here. It was reported that the *pub13* mutant exhibited spontaneous cell death and H_2_O_2_ accumulation under the growth condition of 22 °C, 70% relative humidity, and 250 μE m^–2^ s^–1^ light with a photoperiod of 16h light/8h dark. High humidity treatment (95% relative humidity for 48h) further aggravated these phenotypes (Li *et al.*, 2012). Notably, 250 μE m^–2^ s^–1^ light is a relatively high intensity for *Arabidopsis* growth. When grown under the condition of 23 °C, 65% relative humidity, 75 μE m^–2^ s^–1^ light, with a photoperiod of 12h light/12h dark, neither *pub13* nor *pub12/13* mutant plants exhibited detectable cell death and H_2_O_2_ accumulation (Fig. 5D, E). The plant defence hormone SA was indicated to be involved in PUB13-regulated defence (Li *et al.*, 2012). Endogenous SA levels were measured in WT and PUB13ARM transgenic plants without and with *Pst* DC3000 infection, and no statistically significant difference was detected between WT and PUB13ARM transgenic plants (Fig. 5F). In addition, the transcript level of *PR1*, a marker gene in the SA signalling pathway, and *PDF1.2*, a marker gene in the ethylene (ET) and jasmonic acid (JA) signalling pathways, remained similar among WT, *pub12/13* mutant, and PUB13ARM transgenic plants under the growth conditions used (Fig. 5G). In addition, the flg22-induced FLS2 degradation was comparable in the WT and the *sid2* mutant deficient in SA biosynthesis (Supplementary Fig. S5A at *JXB* online), suggesting that flg22-induced FLS2 degradation is largely SA independent. This is further substantiated by the earlier observations that *eds1*, *npr1*, and *pad4* mutants deficient in SA signalling and plants expressing *NahG* (a salicylate hydroxylase) show normal flg22-mediated resistance (Zipfel *et al.*, 2004). Furthermore, the flg22-induced MAPK activation and ROS production were also elevated in the *sid2pub13* mutant compared with the *sid2* mutant (Supplementary Fig. S5B, C). Taken together, the data indicate that flg22-induced PUB13-mediated early defence responses are not completely due to the elevated SA level in the *pub13* mutant.

### PUB12 and PUB13 negatively regulate stress-induced leaf senescence

**Fig. 6.**
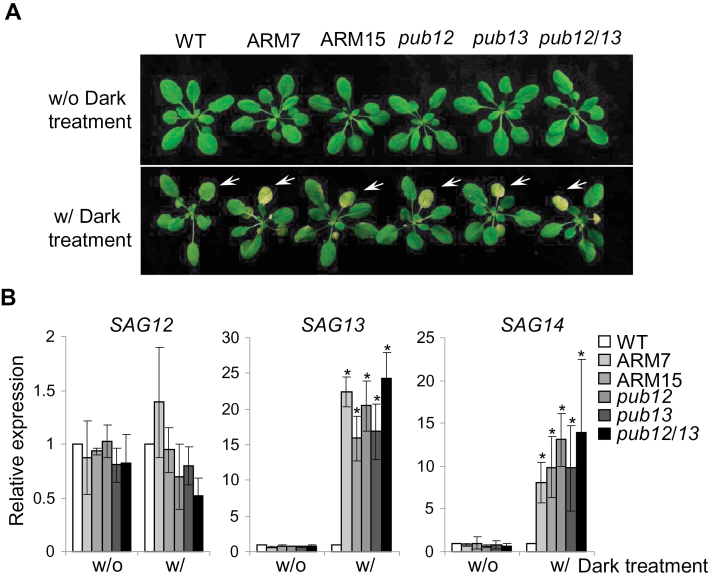
PUB12/13 negatively regulate leaf senescence. (A) Dark-induced senescence in *pub* mutant and ARM transgenic plants. Four-week-old WT, *pub* mutants, and PUB13ARM transgenic plants were subjected to a 4 d dark treatment. Yellowing leaves at the same positions are indicated by the white-coloured arrows. (B) Expression of *senescence-associated genes* (*SAG*) in WT, *pub* mutants, and PUB13ARM transgenic plants. The expression of *SAG12*, *SAG13*, and *SAG14* was normalized to the expression of *UBQ10*. The data are shown as the mean ±SE (*n*=3), and the asterisks (*) indicate a significant difference compared with WT plants (*P*<0.05). The above experiments were repeated three times with similar results.

The phenotypes of *pub12/13* mutant and PUB13ARM transgenic plants were not obviously different from those of WT plants at the 4-week-old stage in the growth conditions used here (Fig. 6A). However, it was noticed that mutant and transgenic plants were prone to be stressed. After a 4 d period of dark treatment, *pub12* and *pub13* single mutant or *pub12/13* double mutant plants senescenced earlier with more yellowing leaves than WT plants (Fig. 6A). Thus, it appears that stresses, including increased light intensity, high humidity, and dark treatment, probably induce cell death and/or H_2_O_2_ accumulation in *pub13* and *pub12* mutant plants. These observations indicate that PUB12 and PUB13 may play a role in stress-induced senescence and cell death regulation. To test this, the transcript levels of several SAGs were measured, among which *SAG12* is a commonly used marker for age-dependent leaf senescence whereas *SAG13* and *SAG14* are stress-induced senescence markers (Du *et al.*, 2013). Importantly, the *pub12*, *pub13*, and *pub12/13* mutants exhibited a WT level of *SAG12* expression whereas they showed dramatically enhanced transcript levels of *SAG13* and *SAG14* upon dark treatment (Fig. 6B). The data suggest that PUB12 and PUB13 are involved in stress-induced leaf senescence regulation. Similar to the *pub12/13* double mutant, PUB13ARM transgenic plants also showed early senescence with elevated transcript levels of *SAG13* and *SAG14* after dark treatment (Fig. 6A, B). To determine the role of SA in PUB13-regulated leaf senescence, *sid2* and *sid2pub13* mutants were subjected to dark treatment. Similar to *pub13*, the *sid2pub13* mutant, but not *sid2*, showed yellowing leaves after 4 d dark treatment (Supplementary Fig. S6A at *JXB* online), indicating that PUB13-mediated senescence is SA independent.

### PUB13ARM transgenic plants show early flowering

**Fig. 7.**
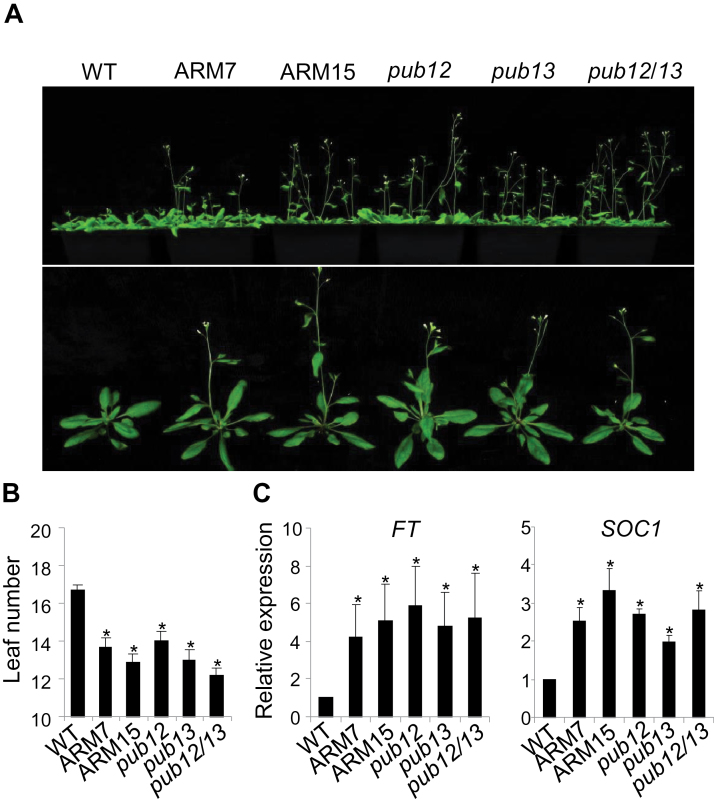
PUB12/13 negatively regulate plant flowering time. (A) Flowering phenotype. Photos were taken of 8-week-old WT, *pub* mutants, and PUB13ARM transgenic plants grown under 23 °C, 65% relative humidity, 75 μE m^–2^ s^–1^ light, and a photoperiod of 16h light/8h dark. (B) Leaf number of WT, *pub* mutants, and PUB13ARM transgenic plants. The leaf number of the tested plants was counted when the first flower bud appeared. An asterisk (*) indicates a significant difference (*P*<0.05) compared with the leaf number of WT plants. (C) Expression of flowering marker genes in the WT, *pub* mutants, and PUB13ARM transgenic plants. The expression of *FT* and *SOC1* was normalized to the expression of *UBQ10*. The data are shown as the mean ±SE (*n*=3), and the asterisks (*) indicate a significant difference compared with WT plants (*P*<0.05). The above experiments were repeated three times with similar results.

The *pub13* mutant displayed early flowering compared with WT plants under 12h light/12h dark or 16h light/8h dark (LD) photoperiods (Li *et al.*, 2012). Similar to the *pub13* mutant, *pub12* and *pub12/13* mutant plants and PUB13ARM transgenic plants flowered ~3 d earlier than the WT (Fig. 7A). The rosette leaf number at flowering of two lines of PUB13ARM transgenic plants was three to four less than that of WT plants (Fig. 7B). Signal transduction pathways regulating flowering time converge at the integrators including flowering locus T (FT), suppressor of overexpression of constans 1 (SOC1), and LEAFY (LFY), and the expression level of these integrators determines the exact flowering time (Seo *et al.*, 2009). qRT–PCR analysis indicated that the transcript levels were elevated by ~4-fold for *FT* and 2-fold for *SOC1* in PUB13ARM transgenic plants and the *pub12/13* mutant compared with WT plants (Fig. 7C). The flowering time of the WT, and *pub13*, *sid2*, and *sid2pub13* mutants was further compared. Although *pub13* mutant plants flowered at 8 weeks post-germination, earlier than WT plants, the *sid2* mutant flowered later than WT plants (Supplementary Fig. S6B) as reported (Martinez *et al.*, 2004). However, both the WT and the *sid2pub13* mutant flowered at ~9 weeks post-germination, which was earlier than the *sid2* mutant (Supplementary Fig. S6C). Therefore, early flowering was still observed in the *sid2pub13* mutant compared with the *sid2* mutant.

## Discussion

The ARM repeat domain, composed of multiple tandem repeats of an ~40 amino acid motif, was first discovered in *Drosophila* β-catenin, and later was found in all eukaryotic organisms (Samuel *et al.*, 2006). The *Arabidopsis* genome encodes ~94 ARM repeat-containing proteins, 41 of which belong to the PUB family (Samuel *et al.*, 2006). Although the ARM repeat domain is predicted to be involved in protein–protein interactions, few interacting proteins have been identified for plant ARM repeat proteins. In this study, it is shown that the kinase domain of the receptor-like kinase BAK1 physically interacts with the ARM domain of PUB13 *in vivo* and *in vitro*. This interaction is specific since the PUB13 ARM domain does not interact directly with the FLS2 kinase domain. Interestingly, the interaction with BAK1 for the ARM domain of PUB13 is much stronger than that for the full-length PUB13 (Fig. 1B and 1C), suggesting that other region(s) of PUB13 may negatively regulate the ARM–BAK1 interaction. PUB13 and some other PUBs have a plant-specific N-terminal UND with unknown functions. It is possible that the UND regulates ARM–BAK1 interaction. BAK1 interacts and phosphorylates the PUB13 ARM domain. It was attempted to define BAK1 phosphorylation motif/residues in the ARM domain with truncations of individual ARM repeats. It appears that BAK1 probably phosphorylates the PUB13 ARM domain at multiple sites and could strongly phosphorylate each individual ARM repeat (data not shown). Consistently, mass spectrometry analysis identified many phosphorylation sites scattered in the ARM domain by BAK1 (data not shown). Thus, it is challenging to address the consequence of BAK1 phosphorylation on PUB13 with mutational analysis. It was shown previously that BAK1 is required for flg22-induced FLS2–PUB13 association and FLS2 degradation, and the kinase inhibitor could block flg22-induced FLS2–PUB13 association (Lu *et al.*, 2011). It is likely that PUB13 phosphorylation by BAK1 leads to its association with FLS2 for receptor ubiquitination and degradation.

**Fig. 8.**
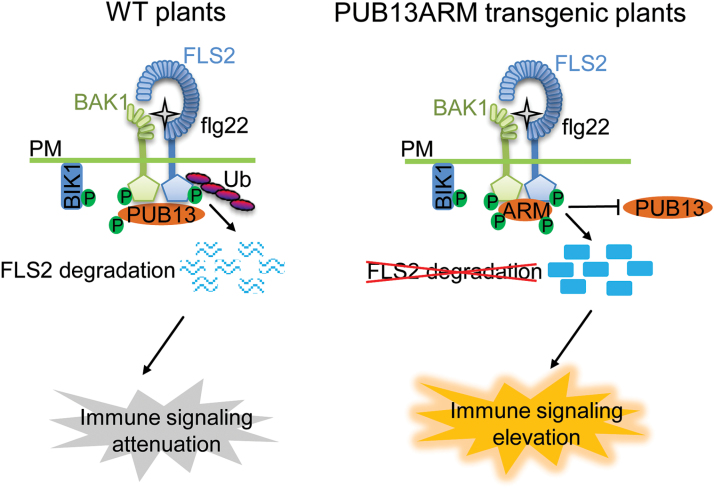
A model of dominant negative function of ectopic expression of the PUB13 ARM domain in plant immunity. In WT plants, perception of flg22 by FLS2 induces formation of the FLS2–BAK1 complex, release of BIK1, and activation of downstream immune responses. The ARM domain of PUB13 interacts with BAK1 and is phosphorylated by BAK1. PUB13 associates with FLS2 during flagellin-induced formation of the FLS2–BAK1 complex, mediates the ubiquitination of FLS2, and directs FLS2 for degradation, resulting in the attenuation of immune signalling. In the PUB13ARM transgenic plants, the overexpressed PUB13ARM proteins strongly interact with BAK1 and prevent BAK1 interaction with endogenous PUB13 and probably PUB12, or alternatively interfere with the function of endogenous PUB13 and PUB12. Therefore, the ectopically expressed PUB13ARM proteins block PUB12/13-mediated FLS2 ubiquitination and degradation, resulting in elevated immune responses. P, phosphate; Ub, ubiquitin; PM, plasma membrane.

Among 64 *Arabidopsis* PUB proteins, 41 of them contain the ARM repeat domain. The functions of the majority of ARM domain-containing PUB proteins have not been elucidated (Yee and Goring, 2009). This might be due in part to the functional redundancy of closely related PUB protein family members. For example, three ARM-containing *Arabidopsis* PUBs, PUB22, PUB23, and PUB24, play a redundant role in PTI signalling (Trujillo *et al.*, 2008), and PUB22 and PUB23 also have a combinatory function in response to drought stress (Cho *et al.*, 2008). PUB12 and PUB13 with 65% amino acid identity and 79% similarity possess functional redundancy in fine-tuning flg22-mediated immune responses (Lu *et al.*, 2011). Notably, PUB12/13 and PUB22/23/24 target different components in FLS2 signalling, suggesting that ubiquitination regulates multiple steps in plant innate immunity. Nevertheless, there is a pressing need for the generation of PUB mutants with the possibility to block the functions of multiple closely related members. In this study, it was shown that ectopic expression of the ARM domain of PUB13 acts as a dominant negative mutant that interferes with activities of endogenous PUB12 and PUB13 proteins. The PUB13 ARM domain strongly interacts with BAK1, thereby blocking BAK1 interaction and phosphorylation of endogenous PUB12 and PUB13. Alternatively, the PUB13 ARM domain may directly interfere with the function of endogenous PUB12 and PUB13. This leads to the attenuation of PUB12/13–FLS2 association and PUB12/13-mediated FLS2 ubiquitination and degradation (Fig. 8). Consistent with this hypothesis, stable transgenic plants overexpressing the PUB13 ARM domain resemble the *pub12/13* double mutant with elevated immunity-related gene expression, ROS production, and callose deposition upon flg22 treatment and enhanced resistance to bacterial infections compared with WT plants (Fig. 3). It was further demonstrated here that both PUB13ARM transgenic plants and *pub12/13* mutant plants showed enhanced MAPK activation upon flg22 treatment (Fig. 4), consistent with the regulation of the upstream FLS2 receptor by PUB12 and PUB13. Thus, ectopic expression of the ARM domain could provide an alternative approach to study the complex function of PUBs and other ARM domain-containing proteins.

The phenotypic resemblance between PUB13ARM transgenic plants and *pub12/13* mutant plants extends to the regulation of flowering. Both plants showed early flowering and elevated expression of flowering marker genes compared with WT plants (Fig. 7). It has been shown that SPL11, the rice orthologue of *Arabidopsis* PUB13, regulates flowering via interaction with SPIN1, a K homology domain protein involved in RNA binding (Vega-Sanchez *et al.*, 2008). SPIN1 represses flowering since overexpression of SPIN1 caused late flowering independently of daylength (Vega-Sanchez *et al.*, 2008). In contrast to the *Arabidopsis pub13* mutant with an early flowering phenotype (Li *et al.*, 2012) (Fig. 7), the rice *spl11* mutant showed delayed flowering under LD conditions (Vega-Sanchez *et al.*, 2008). SPL11 monoubiquitinated SPIN1; however, rather than controlling SPIN1 protein stability, SPL11 regulates SPIN1 mainly at the transcriptional level (Vega-Sanchez *et al.*, 2008). The results indicate that a different mechanism may exist for PUB13 regulation of *Arabidopsis* flowering time. Ubiquitination has been reported to modulate flowering time by regulating the stability and accumulation of CONSTANS (CO; a photoperiodic protein), GIGANTEA (GI; a circadian-associated protein), and FLC (a negative regulator of the autonomous and vernalization pathways) (Liu *et al.*, 2012). PUB13 positively regulates the flowering suppressor FLC to delay flowering under LD conditions (Liu *et al.*, 2012). However, the detailed mechanism of how PUB13 regulates FLC is unknown. Although immunity and flowering are two seemingly independent pathways in plants, emerging evidence suggests that the two pathways are probably interconnected. Similar to the dual functions of PUB13 in immunity and flowering, the *Arabidopsis* small ubiquitin-like modifier (SUMO) E3 ligase SIZ1 is a negative regulator of both immunity and flowering time (Lee *et al.*, 2007; Jin *et al.*, 2008). HopW1-1-interacting 3 (WIN3) was reported to promote plant innate immunity and delay flowering time in *Arabidopsis* (Wang *et al.*, 2011). These findings suggest that tightly controlled flowering time may associate with plant fitness to defend against pathogen attacks.

PUB13ARM transgenic plants and *pub12/13* mutant plants are also more sensitive to dark-induced senescence with elevated transcript levels of stress-induced senescence marker genes (Fig. 6). Leaf senescence is a type of PCD that allows plants to mobilize nutrients from senescing cells to actively growing tissues (Zhang *et al.*, 2013). Another ARM domain-containing PUB protein, PUB44, also regulates leaf senescence. The *Arabidopsis pub44* mutant, also termed *senescence-associated ubiquitin ligase1* (*saul1*), showed early senescence when challenged with low light (20 μE m^–2^ s^–1^) (Vogelmann *et al.*, 2012). Interestingly, *saul1/pub44* mutation-mediated senescence depends on the PAD4-dependent and NPR1-independent SA pathway. Accumulating evidence has suggested that plant defence and leaf senescence share some components in SA signalling and regulation. An SA catabolic enzyme SA 3-hydroxylase regulates onset and progression of leaf senescence (Zhang *et al.*, 2013). Several SAGs have been identified as defence-related genes, such as *SAG26*, *SAG29*, *elicitor-activated gene 3-2* (*ELI3-2*), *nitrilase 2* (*NIT2*), *osmotin* 34 (*AtOSM34*), *sucrose-induced receptor kinase 1* (*SIRK1*), and *WRKY6* (Quirino *et al.*, 1999; Robatzek and Somssich, 2002). It has been shown that mutation of a leucine-rich repeat RLK, RPK1, resulted in significant delay in age- and stress hormone abscisic acid-induced senescence (Lee *et al.*, 2011). It will be interesting to test whether PUB13 and/or PUB44 control leaf senescence through regulating RPK1 or other above-mentioned proteins by ubiquitination-mediated degradation.

## Supplemtentary data

Supplemtental data ara available at *JXB* online.

Figure S1. PUB12ARM and PUB13ARM interact with BAK1 in Co-IP assays.

Figure S2. PUB13 is phosphorylated by BAK1.

Figure S3. Confirmation of PUB13ARM transgenic plants.

Figure S4. PUB12/13 negatively regulate MAPK activation.

Figure S5. Flg22-induced immune responses in the WT, and *pub13*, *sid2*, and *sid2pub13* mutants.

Figure S6. Dark-induced senescence and flowering time of the WT, and *pub13*, *sid2*, and *sid2pub13* mutants.

Table S1. Primers used for cloning and RT–PCR.

## Supplementary Material

Supplementary Data
