# Supplementary material for: The dominant negative ARM domain uncovers multiple functions of PUB13 in Arabidopsis immunity, flowering, and senescence
Source: J Exp Bot. 2015 Apr 11;66(11):3353–66. doi: 10.1093/jxb/erv148 (PMC4449551; doi:10.1093/jxb/erv148)
Supplement: Supplementary Data [file supp_66_11_3353__index.html]

The dominant negative ARM domain uncovers multiple functions of PUB13 in Arabidopsis immunity, flowering, and senescence — The dominant negative ARM domain uncovers multiple functions of PUB13 in Arabidopsis immunity, flowering, and senescence — Supplementary Data 

# The dominant negative ARM domain uncovers multiple functions of PUB13 in *Arabidopsis* immunity, flowering, and senescence

## Supplementary Data

Data files

**Files in this Data Supplement:**

- Supplementary Data - Supplementary Data
