# Supplementary material for: The dominant negative ARM domain uncovers multiple functions of PUB13 in Arabidopsis immunity, flowering, and senescence
Source: J Exp Bot. 2015 Apr 11;66(11):3353–66. doi: 10.1093/jxb/erv148 (PMC4449551; doi:10.1093/jxb/erv148)
Supplement: Supplementary Data [file supp_erv148_jexbot146308_file001.pdf]

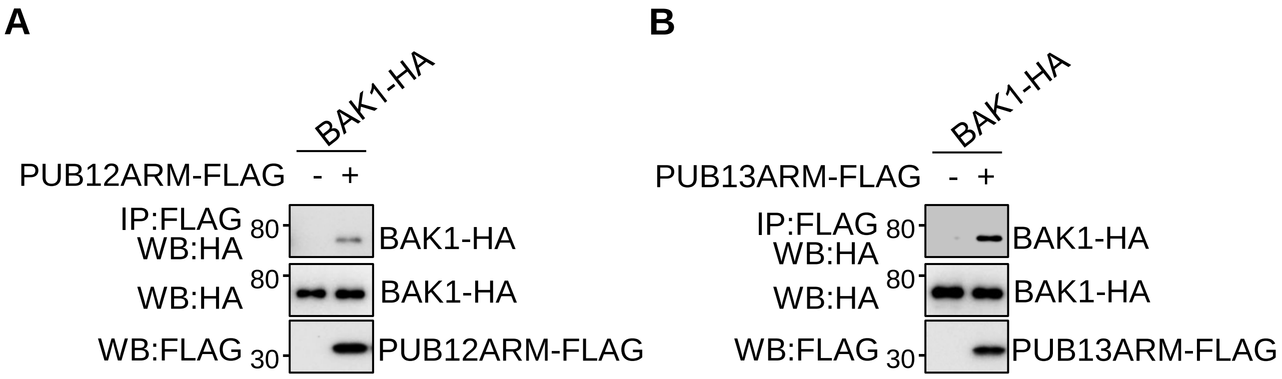

Figure S1. PUB12ARM and PUB13ARM interact with BAK1 in Co-IP assays.
Arabidopsis protoplasts were transfected with BAK1-HA and PUB12ARM-FLAG **(A)**, PUB13ARM-FLAG **(B)** or a control vector. The association of BAK1 with PUBs was detected by α-HA Western blots after α-FLAG immunoprecipitation. The protein levels of BAK1, PUB12ARM and PUB13ARM were detected by α-HA or α-FLAG Western blot respectively.

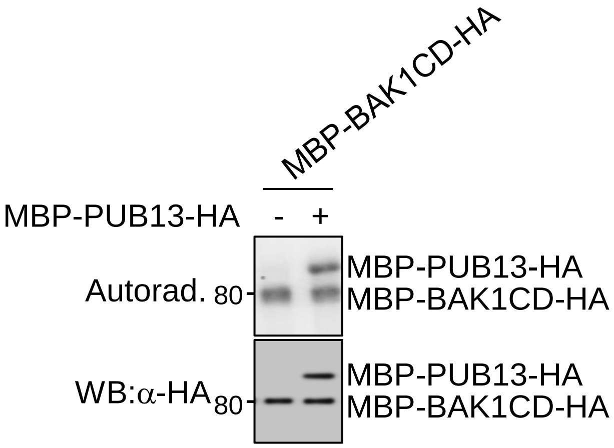

Figure S2. PUB13 is phosphorylated by BAK1.
MBP-PUB13-HA protein (1μg) was used as substrates and MBP-BAK1CD-HA (1μg) as the kinase in an *in vitro* kinase assay. Phosphorylation was detected by autoradiography (top panel), and the protein loading is shown by an α-HA Western blot (bottom panel).

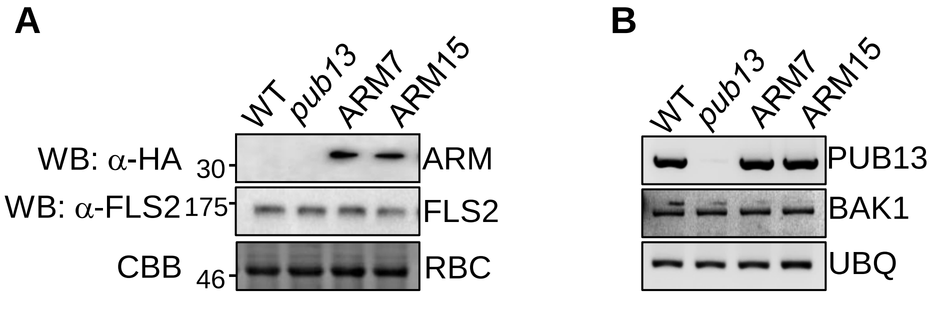

Figure S3. Confirmation of PUB13ARM transgenic plants.
(A) Expression of PUB13ARM proteins in the PUB13ARM transgenic plants. Protein levels of PUB13ARM and endogenous FLS2 were detected by Western blot with an α-HA or α-FLS2 antibody respectively. The protein loading is shown by CBB for RBC. **(B)** Intact PUB13 genomic DNA in the PUB13ARM transgenic plants. The genomic DNA of *PUB13* and *BAK1* was determined by PCR. *UBQ10* was used as a control for loading.

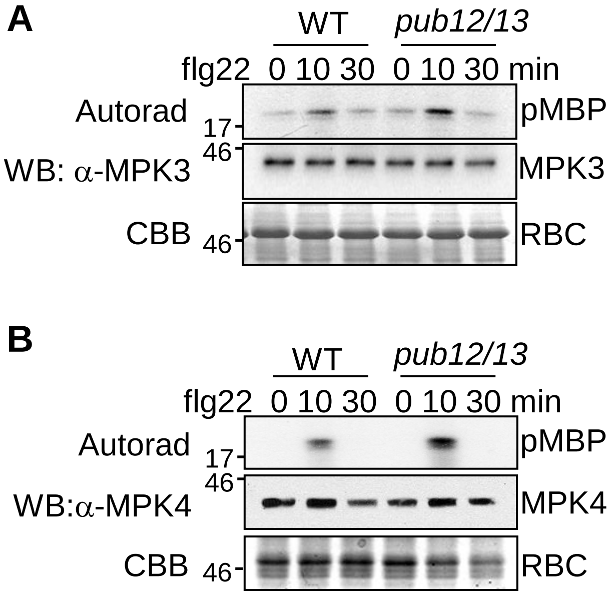

Figure S4. PUB12/13 negatively regulate MAPK activation.
(A) & **(B)** Enhanced MAPK activity in the *pub12/13* mutant as detected by immunocomplex kinase assay. Two-week-old seedlings were treated with 1 μM flg22 for 10 or 30 min. MPK3 (A) and MPK4 (B) proteins were immunoprecipitated with α-MPK3 or α-MPK4 antibody respectively and subjected to an *in vitro* kinase assay using MBP as the substrate. The protein loading of MPK3 or MPK4 is shown by α-MPK3 or α-MPK4 Western blot.

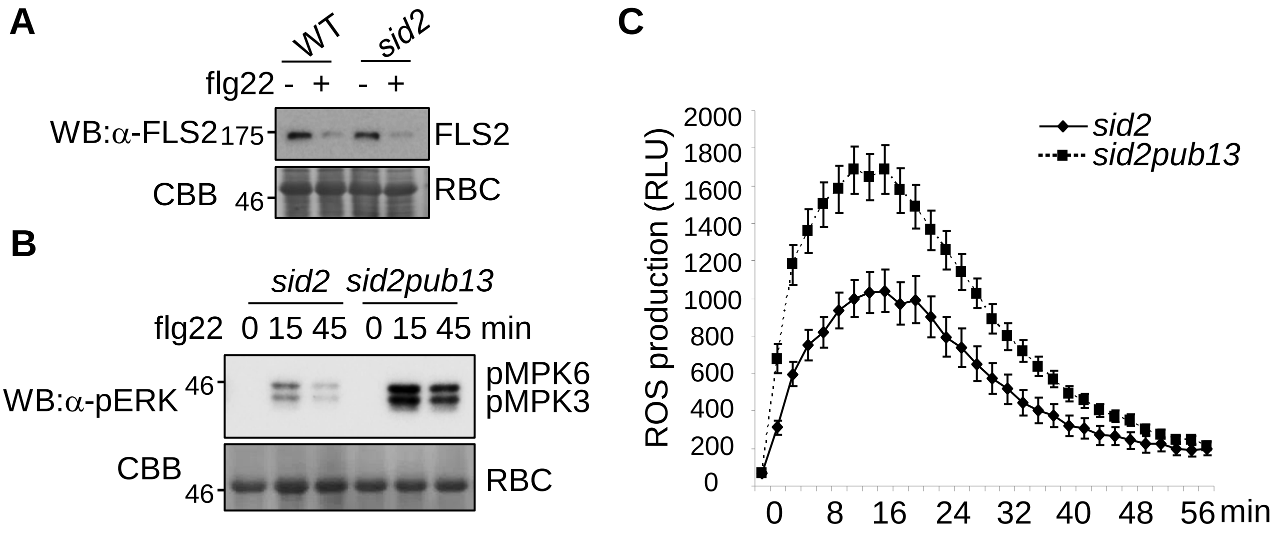

Figure S5. Flg22-induced immune responses in WT, *pub13*, *sid2* and *sid2pub13* mutants.

(A) Flg22-induced FLS2 degradation in WT and *sid2* mutant plants. Two-week-old seedlings were treated with 1 μM flg22 for1 hr. The endogenous FLS2 proteins were detected by α-FLS2 Western blot and the equal protein loading is shown by CBB staining for RuBisCo (RBC). **(B)** MAPK activity in *sid2* and *sid2pub13* mutants as detected by α-pERK antibody. Two-week-old seedlings were treated with 100 nM flg22 for 15 or 45 min. Phosphorylated MPK3 (pMPK3) and MPK6 (pMPK6) were detected by α-pERK Western blot. The protein loading is shown by CBB for RBC. **(C)** ROS production in *sid2* and *sid2pub13* mutants. Leaf discs from five-week-old plants were treated with 100 nM flg22 and ROS production was detected at indicated time points. The data are shown as the mean ± standard error from 24 leaf discs.

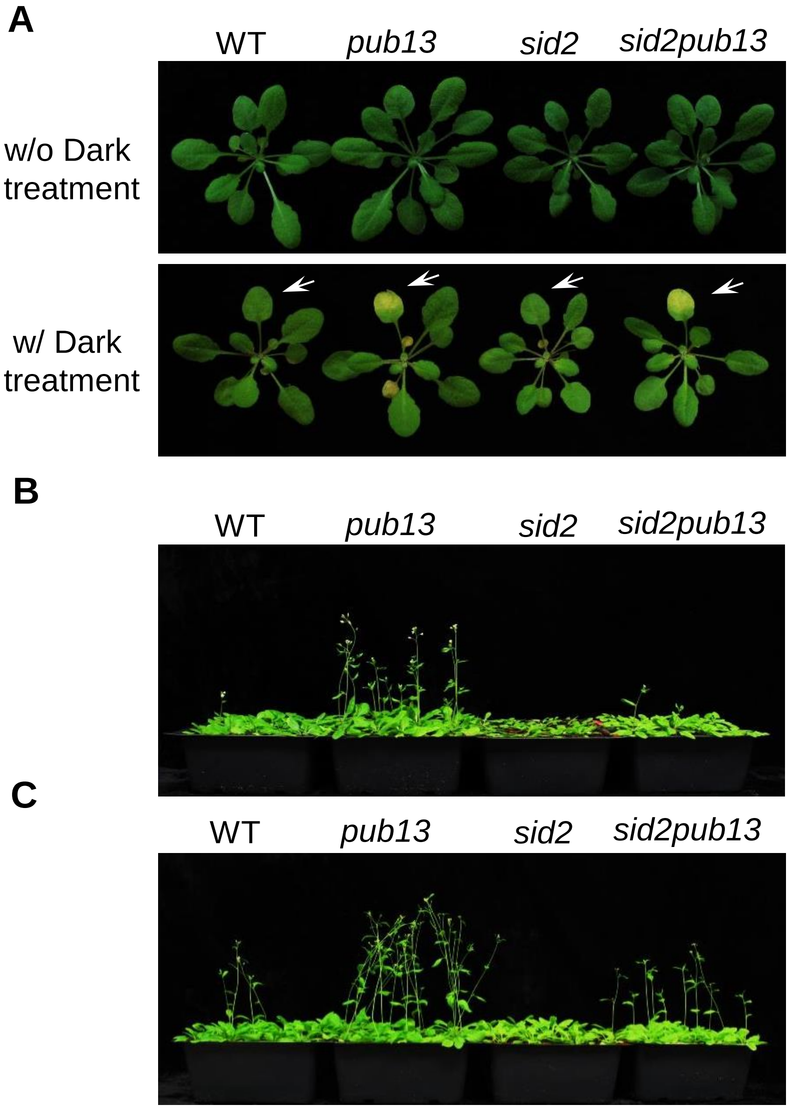

Figure S6. Dark-induced senescence and flowering time of WT, *pub13*, *sid2* and *sid2pub13* mutants.

(A) Dark-induced leaf senescence of plants. Four-week-old plants were subjected to a four-day dark treatment. Yellowing leaves at the same positions are indicated by the white-colored arrows. **(B)** and **(C)** Flowering phenotype of plants. Photos were taken for eight-week-old (B) and nine-week-old (C) plants grown under 23°C, 65% relative humidity, 75 μE m^{-2} s^{-1} light and photoperiod of 16 hr of daylight /8 hr of dark.

Primers for cloning and PCR analysis (the restriction enzyme sites are underlined and start codon is italicized):

PUB13 UND-F	5'-CATGCC*ATG*GAGGAAGAGAAAGCTTC-3'
PUB13 UND-R	5'-GAAGGCCTTATCTTCTGACTCGCTGCC-3'
PUB13 U-box-F	5'-CATGCC*ATG*GCGAGTCAGAAGATACCTGTG-3'
PUB13 U-box-R	5'-GAAGGCCTCTCAATATCGTTGGCCTCGC-3'
PUB13 ARM-F	5'-CATGCC*ATG*GAGCCTCCAAAGCCTCCGAG-3'
PUB13 ARM-R	5'-GAAGGCCTAGTATCTGCAGCTTCTGTGG-3'
PUB12 ARM-F	5'-CATGCC*ATG*GAGCCTCCAAAGCGTCCCAAC-3'
PUB12 ARM-R	5'-TCCCCCGGGGATTAGGGAGATTTGATCTTCC-3'
BAK1-F	5'-CATGCC*ATG*GAACGAA GATTAATGATC-3'
BAK1-R	5'-GAAGGCCTTCTTGGACCCGAGGGGTATTC-3'

Primers for RT-PCR analysis:

WRKY30-F	5'-GCAGCTTGAGAGCAAGAATG-3'
WRKY30-R	5'-AGCCAAATTTCCAAGAGGAT-3'
AP2-F	5'-CTCAGCGGTCTCAAATGTCC-3'
AP2-R	5'-AGGAGCAGCAACAACCAATC-3'
FRK1-F	5'-ATCTTCGCTTGGAGCTTCTC-3'
FRK1-R	5'-TGCAGCGCAAGGACTAGAG-3'
SAG12-F	5'-CTGCGAAGGCGGTTTAATGG-3'
SAG12-R	5'-CGGGACATCCTCATAACCTGT-3'
SAG13-F	5'-CTTACGTGAATGGCAAGCAA-3'
SAG13-R	5'-CCACATTGTTGACGAGGATG-3'
SAG14-F	5'-CCCAAGTACTGGAGGAACCA-3'
SAG14-R	5'-TAATGAGGAAGCGGCATTTC-3'
PR1-F	5'-ACACGTGCAATGGAGTTTGTGG-3'
PR1-R	5'-TTGGCACATCCGAGTCTCACTG-3'
PDF1.2-F	5'-TGTTTGGCTCCTTCAAGGTT-3'
PDF1.2-R	5'-TTCTCTTTGCTGCTTTCGAC-3'
UBQ10-F	5'-AGATCCAGGACAAGGAAGGTATTC-3'
UBQ10-R	5'-CGCAGGACCAAGTGAAGAGTAG-3'

Table S1
